# Exercise Prescription in Individuals with Prehypertension and Hypertension: Systematic Review and Meta-Analysis

**DOI:** 10.31083/j.rcm2504117

**Published:** 2024-03-27

**Authors:** Yang Xi, Xiaoyun Liu, Yuanyuan Chen

**Affiliations:** ^1^Department of Hypertension, Peking University People’s Hospital, 100044 Beijing, China; ^2^Department of Phase I Clinical Trial Center, Beijing Shijitan Hospital, Capital Medical University, 100038 Beijing, China

**Keywords:** exercise prescription, blood pressure, heart rate, hypertension, prehypertension

## Abstract

**Background:**

The prevalence of prehypertension and hypertension has been increasing over the years, and is closely related to cardiovascular and cerebrovascular diseases. Exercise is an effective method of lifestyle intervention, and it aims to lower blood pressure and control other risks. Studies have shown that different modes of exercise have varying effects on blood pressure, and individuals with prehypertension or hypertension need to carry out this intervention by using personalized modes of exercise.

**Methods:**

We conducted a systematic review and meta-analysis to evaluate the effects of different modes of exercise regimens on systolic blood pressure, diastolic blood pressure and heart rate in individuals with high-normal blood pressure and hypertension. We included 27 trials, and 2731 individuals were under 8 exercise regimens. Stata12.0 statistical software was used for statistical analysis.

**Results:**

Heat pools significantly reduced systolic blood pressure (SBP) by 15.62 mmHg (95% confidence interval [CI]: –23.83, –7.41), and cycling reduced SBP by 14.76 mmHg (–17.04, –12.48). Two to three types of aerobic exercise performed at the same time also significantly reduced diastolic blood pressure (DBP) by 5.61 mmHg (–7.71, –3.52), and isometric handgrip training exercise reduced DBP by 5.57 mmHg (–7.48, –3.66). Cycling also significantly reduced heart rate (HR) by 9.57 beats/minute (–11.25, –7.90).

**Conclusions:**

The existing literature suggests that different types of exercise can effectively reduce the levels of SBP, DBP and HR in individuals with prehypertension or hypertension.

## 1. Introduction

In recent years, the prevalence of prehypertension and hypertension has generally been increasing, and is closely related to cardiovascular and cerebrovascular diseases [1, 2, 3]. Lifestyle intervention is considered as a rational and effective method at any time for any individual (including those with prehypertension and those with hypertension requiring drug treatment) because it aims to lower blood pressure (BP) and control other risks. Exercise is an effective method of lifestyle intervention. To date, related USA and Chinese guidelines on exercise have proposed relevant suggestions. The Chinese Guideline on Healthy Lifestyle to Prevent Cardiometabolic Diseases suggests that, if the physical condition allows, exercise can be increased to 300 minutes of moderately intensive exercise or 150 minutes of high-intensity aerobic exercise each week, or an equivalent combination of two intensive exercises [4, 5].

Studies have shown that different modes of exercise have varying effects on BP, and people with prehypertension or hypertension need to carry out this intervention using personalized modes of exercise. To date, Chinese and international hypertension guidelines emphasize that a lifestyle intervention should be adopted for individuals with prehypertension or hypertension, and state that regular exercise is a crucial factor [6, 7]. This study aimed to systematically review the relevant literature to provide relevant statistics on different exercise types used in different populations according to the individual BP level, and preferably select accessible and optimal exercises with anti-hypertensive effects.

## 2. Materials and Methods

### 2.1 Data Collection

We selected keywords, namely “exercise”, “physical activity”, “training”, “sports”, “hypertension”, “prehypertension”, and “high-normal blood pressure”, and retrieved them on the EMBASE website (including EMBASE and MEDLINE databases). In addition, strict inclusion and exclusion criteria were applied to select literature that satisfied the method of references, including review retrospection, manual retrieval, and primary research tracking, and relevant English literature published from 2000 to 2020.

According to the PRISMA standard [8], we performed a systematic review and meta-analysis regarding randomized, controlled trials (RCTs) of different modes of exercise intervention that targeted prehypertensive and hypertensive individuals, and the last search was updated on 19 February 2021.

The literature search strategy was as follows. Relevant articles were identified by searching https://www.embase.com/ (including EMBASE and MEDLINE) without year and language restrictions, with the following search terms: exercise or physical activity or training or sports; hypertension or prehypertension or blood pressure; meta-analysis or systematic review; and others (see **Supplementary Material**).

### 2.2 Data Analysis

**Fig. 1.**
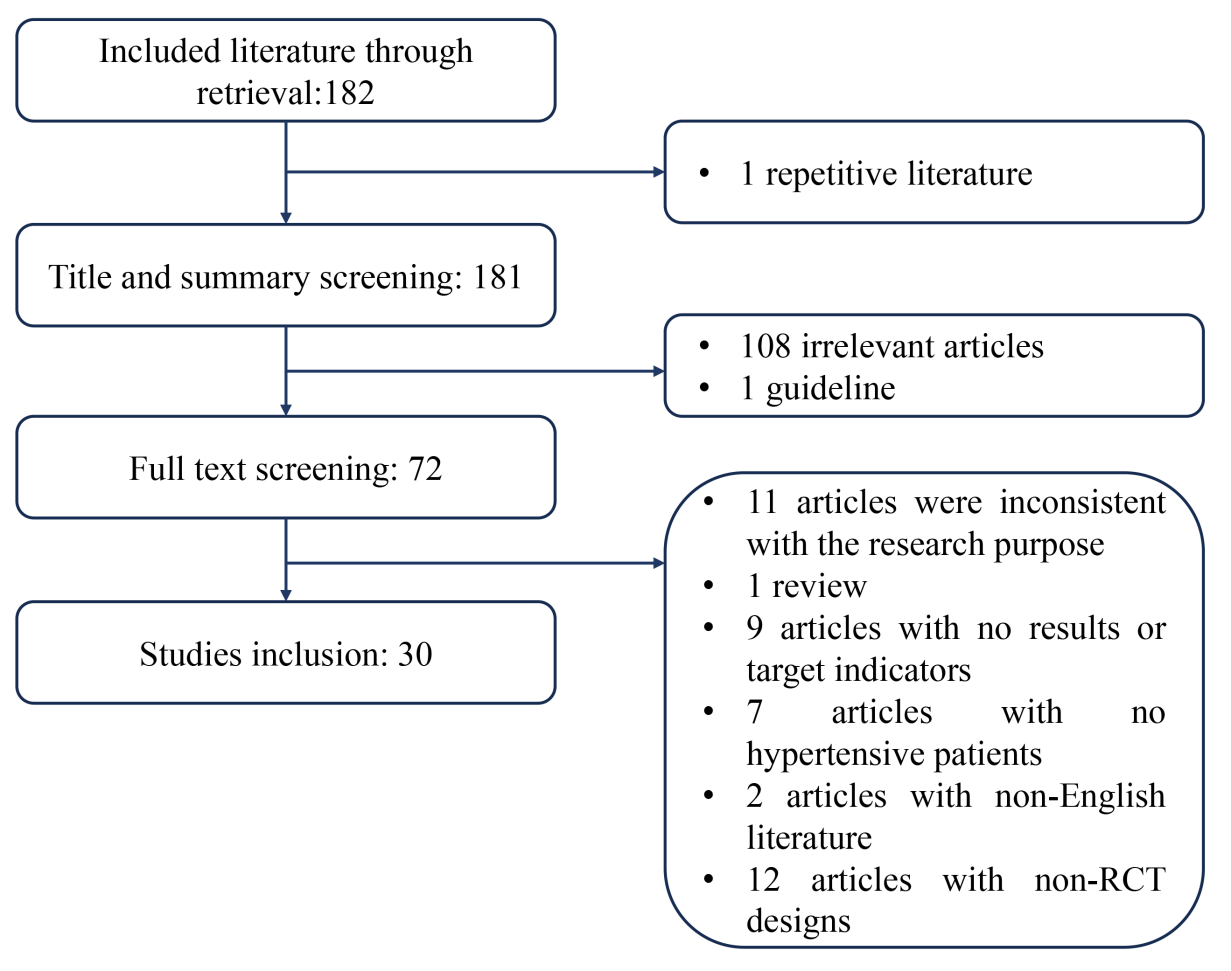
**Flowchart for selecting articles**. RCT, randomized controlled trial.

As a result of the search, 182 articles were included, and 108 irrelevant articles were excluded by manually reviewing the title and abstract, and 1 guideline and 1 repetitive article were excluded after reading their title and abstract. The remaining 72 articles were downloaded and evaluated for eligibility, of which 42 did not meet the inclusion/exclusion criteria. Of these, 11 were inconsistent with the research purpose, 1 was a review, 7 had no results, 2 had no target indicators, 7 included non-hypertensive individuals or those without high-normal BP (including children and adolescents), 2 were non-English articles and 12 were not randomized controlled trial (RCT) designs. Finally, 30 articles were included in the systematic review and meta-analysis. The article screening process is shown in Fig. 1.

### 2.3 Basic Characteristics of the Included Articles

Articles meeting the following criteria were included in the analysis: (1) original RCTs and review articles were not be included, but we checked whether the original research described in review articles was included in the data analysis; (2) the age of individuals was ≥18 years old of either sex; (3) BP levels represented prehypertension (systolic blood pressure [SBP] of 120–139 mmHg or diastolic blood pressure [DBP] of 80–89 mmHg) or hypertension (SBP ≥140 mmHg and/or DBP ≥90 mmHg); and (4) studies contained data on BP levels or a decline in BP levels before and after treatment.

Articles meeting the following criteria were excluded: (1) non-English literature; (2) non-RCT; (3) research subjects only had normal BP; (4) no BP results and conclusion; and (5) review articles.

### 2.4 Research Quality Evaluation

The improved Jadad score [9] was used for quality review. There was almost no publication bias or heterogeneity in the literature (see **Supplementary Table 1** for details).

### 2.5 Statistical Methods

Stata12.0 statistical software (StataCorp LP, College Station, TX, USA) was used for statistical analysis in this study. The mean and 95% confidence interval (CI) were used as the effect size to evaluate the statistical combination of continuous outcome indicators (e.g., BP). $I^{2}$ statistics were used to evaluate the heterogeneity of each meta-analysis and subgroup analysis. If $I^{2}$ was ≥50% or Cochran’s Q test showed *p* > 0.1, it indicated heterogeneity. In case of heterogeneity, we used the DerSimonian and Laird random effect model to combine the outcome indicators, and otherwise, we used the Mantel–Haenszel fixed effect model. A sensitivity analysis was used to detect the stability of the above-mentioned statistical effect values and/or to identify the source of heterogeneity. Therefore, a meta-analysis was repeated for each deleted study to compare the changes of the effect values before and after. Meta-regression was performed to evaluate the effect of grouping variables on the basis of the total effect value (*p* < 0.05 indicates statistical significance). Funnel plots and Kendall rank correlation tests were used to analyze possible publication bias (*p* < 0.05 indicates that there was publication bias).

## 3. Results

Thirty RCTs were included according to the criteria mentioned above. Table 1 (Ref. [10, 11, 12, 13, 14, 15, 16, 17, 18, 19, 20, 21, 22, 23, 24, 25, 26, 27, 28, 29, 30, 31, 32, 33, 34, 35, 36, 37, 38, 39]) shows the main characteristics of RCTs of different exercise modes. Twenty-seven of these RCTs were included in the systematic review and meta-analysis [10, 11, 12, 13, 14, 15, 16, 17, 18, 19, 20, 21, 22, 23, 24, 25, 26, 27, 28, 29, 30, 31, 32, 33, 34, 35, 36, 37, 38, 39]. The other three could not be systematically reviewed and a meta-analysis performed because they were only single RCT research of related sports modes [19, 29, 31]. **Supplementary Table 2** shows the changes in BP and heart rate (HR) under different exercise regimens.

**Table 1.** **Main characteristics of RCTs of different types of exercise**.

Exercise type	Selected population	Exercise frequency, exercise intensity and exercise time	Sample size (n)	Male/Female (n)	Exercise intervention time	Anti-hypertensive drugs usage
Heat pool [33, 34]	resistant HT/post-menopausal HT women	heat pool (32–33.5 °C), 50–60 min, 3 times/week	84	18/66	12 weeks	0–≥3 kinds
Cycling [20, 25, 33]	post-menopausal HT women/essential HT men/never-treated HT	45–60 min, 3 times/week, moderate intensity, 60–79% HRmax	441	376/65	8 weeks–4 months	0–≤1 kinds
IHG [15, 30, 32, 35, 37]	pre-HT/medicated HT/HT	3–5 times/week or 24 consecutive days, four unilateral 2-min isometric contractions at 30% of maximal voluntary contraction, each separated by 1–4 min of rest	539	294/245	24 days–12 weeks	Yes/No
Treadmill [10, 14, 18, 23, 24, 26, 27, 28, 38]	middle-aged HT/unmedicated high normal or stage 1–2, overweight/elderly HT/HT/resistant HT	AIT/MIT, HRmax 90–95%, 3–5 times/week, HRmax 70%, 30–55 min	597	270/327	8 weeks–6 months	Yes/No/≥3 kinds
Walking [11, 13, 21, 22, 36]	newly diagnosed HT/mild to moderate HT age >60/postmenopausal women with high normal or stage 1–2/HT with CVD risk factors	30 min, 5–7 times/week or 3 km/day	576	283/293	6 weeks–6 months	Yes/No
Resistance [38, 39]	middle-aged HT/postmenopausal women with stage 1 HT	3 times/week, 60 min	67	31/36	12 weeks	0–≥1
Tai Chi [16, 36]	HNBP or stage I/HT with CVD risk factors	30–60 min, 2–3 times/week	322	150/172	12 weeks/3 months	Yes/No
2-3 kinds of aerobic exercise in sync [12, 17]	HNBP or stage 1–2 HT	biking + walking (and eventually jogging), 45 min, 3–4 times/week; jogging + walking + calisthenics, mild exercise at AT, 2 times/week	115	65/50	6 months	-
Swim [29]	pre-hypertension or stage 1 HT	15–20 min/day, 3–4 times/week; 40–45 min/day, 3–4 times/week	43	11/32	12 weeks	0
NIH consensus development panel recommended physical activity dose (on a cycle ergometer) [19]	sedentary, post-menopausal overweight or obese women with SBP range 120–159.9 mmHg	50%, 100% or 150% of the NIH consensus development panel recommended physical activity dose, 3–4 times/week	464	0/464	6 months	-
Soccer training [31]	men with mild-to-moderate hypertension	soccer training, 1 h, 2 times/week	33	33/0	6 months	Yes/No

HT, hypertension; IHG, isometric handgrip training; AIT/MIT, aerobic interval training/moderate intensity continuous training; HR, heart rate; CVD, cardiovascular disease; HNBP, high-normal blood pressure; AT, anaerobic threshold; NIH, National Institutes of Health; RCT, randomized controlled trial; SBP, systolic blood pressure.

### 3.1 Treatment Regimen: Heat Pool

In two studies [33, 34], SBP was decreased by 15.62 mmHg (95% CI: –23.83, –7.41) after heat pool. There was no heterogeneity between the studies ($I^{2}$ = 40.7%, *p* = 0.194) (**Supplementary Fig. 1**).

### 3.2 Treatment Regimen: Cycling 

Three studies reported home BP data with cycling programs [20, 25, 33](**Supplementary Fig. 2**). SBP in the experimental groups was decreased by 16.74 mmHg compared with that in the control groups (95% CI: –19.24, –14.24). There was no heterogeneity between the studies ($I^{2}$ = 0.0%, *p* = 0.526) (**Supplementary Fig. 2A**). SBP was decreased by 14.76 mmHg (95% CI: –17.04, –12.48) after cycling, and there was no heterogeneity between the studies ($I^{2}$ = 0.0%, *p* = 0.856) (**Supplementary Fig. 2B**). In the control group, SBP was increased by 2.55 mmHg (95% CI: –0.01, 5.11) at the end of the study period, and there was no heterogeneity between the studies ($I^{2}$ = 0.0%, *p* = 0.976) (**Supplementary Fig. 2C**).

DBP in the experimental groups was decreased by 3.79 mmHg compared with that in the control group (95% CI: –7.82, 0.23), but this was not significant. There was heterogeneity between the studies ($I^{2}$ = 92.3%, *p* < 0.001) (**Supplementary Fig. 2D**). DBP was decreased by 5.07 mmHg (95% CI: –9.17, –0.98) after cycling, and there was heterogeneity between the studies ($I^{2}$ = 88.8%, *p* < 0.001) (**Supplementary Fig. 2E**). In the control group, DBP was decreased by 1.07 mmHg (95% CI: –1.48, –0.66) at the end of the study period, and there was no heterogeneity between the studies ($I^{2}$ = 0.0%, *p* = 1.000) (**Supplementary Fig. 2F**).

HR in the experimental groups was decreased by 7.95 beats/minute compared with that in the control group (95% CI: –11.08, –4.82). There was no heterogeneity between the studies ($I^{2}$ = 0.0%, *p* = 0.535) (**Supplementary Fig. 2G**). HR was decreased by 9.57 beats/minute (95% CI: –11.25, –7.90) after cycling, and there was no heterogeneity between the studies ($I^{2}$ = 42.0%, *p* = 0.178) (**Supplementary Fig. 2H**). In the control group, HR was decreased by 1.61 beats/minute (95% CI: –5.52, 2.31) at the end of the study period, and there was no heterogeneity between the studies ($I^{2}$ = 0.0%, *p* = 0.851) (**Supplementary Fig. 2I**).

### 3.3 Treatment Regimen: Isometric Handgrip Training

Five studies reported isometric handgrip training (IHG) data [15, 30, 32, 35, 37] (**Supplementary Fig. 3**). SBP in the experimental groups was decreased by 3.51 mmHg compared with that in the control group (95% CI: –4.43, –2.59). There was no heterogeneity between the studies ($I^{2}$ = 0.0%, *p* = 0.435) (**Supplementary Fig. 3A**). SBP was decreased by 8.08 mmHg (95% CI: –12.97, –3.20) after IHG training, and there was no heterogeneity between the studies ($I^{2}$ = 62.0%, *p* = 0.032) (**Supplementary Fig. 3B**). In the control group, SBP was decreased by 3.27 (95% CI: –10.22, 3.68) at the end of the study period, and there was no heterogeneity between the studies ($I^{2}$ = 0.0%, *p* = 0.405) (**Supplementary Fig. 3C**). Among them, the regimens of the control groups in the studies by Pagonas *et al*. [35] and Ogbutor *et al*. [37] were different and could not be combined.

DBP in the experimental groups was decreased by 2.52 mmHg compared with that in the control group (95% CI: –2.95, –2.09). There was no heterogeneity among the studies ($I^{2}$ = 0.0%, *p* = 0.913) (**Supplementary Fig. 3D**). Publication bias was observed (Egger’s test *p* = 0.022). DBP was decreased by 5.57 mmHg (95% CI: –7.48, –3.66) after IHG training, and there was no heterogeneity between the studies ($I^{2}$ = 16.9%, *p* = 0.307) (**Supplementary Fig. 3E**). In the control group, DBP was decreased by 0.04 mmHg (95% CI: –5.09, 5.01) at the end of the study period, and there was no heterogeneity between the studies ($I^{2}$ = 0.0%, *p* = 0.787) (**Supplementary Fig. 3F**). Among them, the regimens of the control groups in the studies by Pagonas *et al*. (2017) [35] and Ogbutor *et al*. (2019) [37] were different and could not be combined.

HR in the experimental groups was decreased by 2.87 beats/minute compared with that in the control group (95% CI: –7.70, 1.96), but this was not significant. There was no heterogeneity between the studies ($I^{2}$ = 0.0%, *p* = 0.644) (**Supplementary Fig. 3G**). HR was decreased by 1.37 beats/minute (95% CI: –6.32, 3.58) after IHG training, and there was no heterogeneity between the studies ($I^{2}$ = 0.0%, *p* = 0.934) (**Supplementary Fig. 3H**). In the control group, HR was increased by 1.62 beats/minute (95% CI: –2.64, 5.87) at the end of the study period, and there was no heterogeneity between the studies ($I^{2}$ = 0.0%, *p* = 0.751) (**Supplementary Fig. 3I**).

### 3.4 Treatment Regimen: Treadmill Exercise

Data from nine studies showed that the treadmill exercise group had significantly reduced BP, HR, and 24-hour ambulatory BP compared with the control group [10, 14, 18, 23, 24, 26, 27, 28, 38].

#### 3.4.1 Changes in BP and HR after Treadmill Exercise

**Fig. 2.**
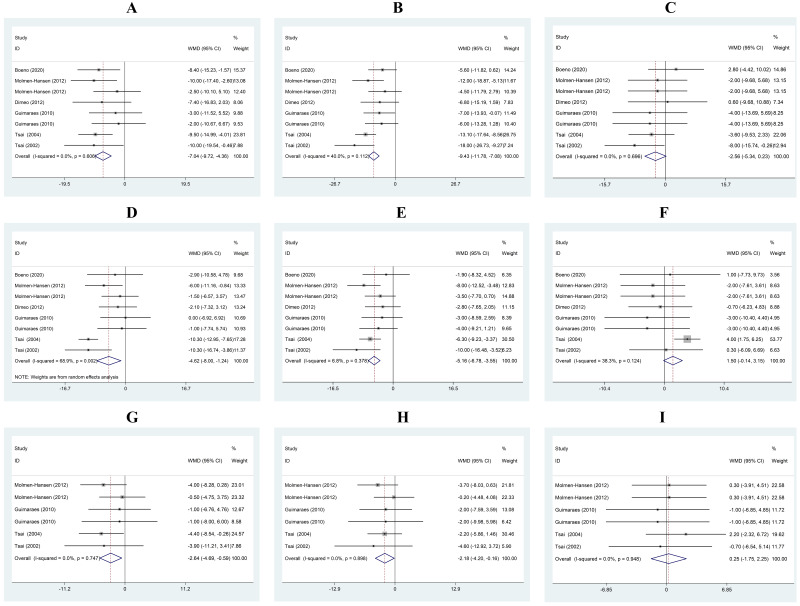
**Mean difference in office BP and HR after treadmill exercise**. (A) Changes in SBP (mmHg) between the experimental and control groups. (B) Changes in SBP (mmHg) in the experimental group (mmHg). (C) Changes in SBP (mmHg) in the control group. (D) Changes in DBP (mmHg) between the experimental and control groups. (E) Changes in DBP (mmHg) in the experimental group (mmHg). (F) Changes in DBP (mmHg) in the control group. (G) Changes in HR (beats/minute) between the experimental and control groups. (H) Changes in HR (beats/minute) in the experimental group. (I) Changes in HR (beats/minute) in the control group. Horizontal lines show 95% CIs with the point estimate at the center of the corresponding box. Within each subplot, boxes are proportional to the sample size from each study. Diamonds represent summary data centered on the pooled estimates, and their width spans the corresponding 95% CIs. CI, confidence interval; WMD, weighted mean difference; BP, blood pressure; DBP, diastolic blood pressure; SBP, systolic blood pressure; HR, heart rate.

SBP in the experimental groups was decreased by 7.04 mmHg compared with that in the control group (95% CI: –9.72, –4.36). There was no heterogeneity between the studies ($I^{2}$ = 0.0%, *p* = 0.606) (Fig. 2A). SBP was decreased by 9.43 mmHg (95% CI: –11.78, –7.08) after treadmill exercise, and there was no heterogeneity between the studies ($I^{2}$ = 40.0%, *p* = 0.112) (Fig. 2B). In the control group, SBP was decreased by 2.56 mmHg (95% CI: –5.34, 0.23) at the end of the study period, and there was no heterogeneity between the studies ($I^{2}$ = 0.0%, *p* = 0.696) (Fig. 2C).

DBP in the experimental groups was decreased by 4.62 compared with that in the control group (95% CI: –8.00, –1.24). There was heterogeneity between the studies ($I^{2}$ = 68.9%, *p* = 0.002) (Fig. 2D). DBP was decreased by 5.16 mmHg (95% CI: –6.78, –3.55) after treadmill exercise, and there was no heterogeneity between the studies ($I^{2}$ = 6.8%, *p* = 0.378) (Fig. 2E). In the control group, DBP was increased by 1.50 mmHg (95% CI: –0.14, 3.15) at the end of the study period, and there was no heterogeneity between the studies ($I^{2}$ = 38.3%, *p* = 0.124) (Fig. 2F).

HR in the experimental groups was decreased by 2.64 beats/minute compared with that in the control group (95% CI: –4.69, –0.59). There was no heterogeneity between the studies ($I^{2}$ = 0.0%, *p* = 0.747) (Fig. 2G). HR was decreased by 2.18 beats/minute (95% CI: –4.20, –0.16) after treadmill exercise, and there was no heterogeneity between the studies ($I^{2}$ = 0.0%, *p* = 0.898) (Fig. 2H). In the control group, HR was increased by 0.25 beats/minute (95% CI: –1.75, 2.25) at the end of the study period, and there was no heterogeneity between the studies ($I^{2}$ = 0.0%, *p* = 0.948) (Fig. 2I). Publication bias was observed (Egger’s test *p* < 0.01).

#### 3.4.2 Changes in 24-Hour Ambulatory BP after Treadmill Exercise

Daytime SBP in the experimental groups was decreased by 6.21 mmHg compared with that in the control group (95% CI: –9.14, –3.29). There was no heterogeneity between the studies ($I^{2}$ = 35.1%, *p* = 0.174) (**Supplementary Fig. 4A**). Daytime SBP was decreased by 5.58 mmHg (95% CI: –8.33, –2.82) after treadmill exercise, and there was no heterogeneity between the studies ($I^{2}$ = 38.9%, *p* = 0.146) (**Supplementary Fig. 4B**). In the control group, daytime SBP was increased by 0.80 mmHg (95% CI: –2.22, 3.82) at the end of the study period, and there was no heterogeneity between the studies ($I^{2}$ = 0.0%, *p* = 0.956) (**Supplementary Fig. 4C**). Daytime DBP in the experimental groups was decreased by 4.61 mmHg compared with the control group (95% CI: –6.43, –1.89). There was no heterogeneity between the studies ($I^{2}$ = 0.0%, *p* = 0.786) (**Supplementary Fig. 4D**). Daytime DBP was decreased by 4.26 mmHg (95% CI: –6.18, –2.34) after treadmill exercise, and there was no heterogeneity between the studies ($I^{2}$ = 0.0%, *p* = 0.479) (**Supplementary Fig. 4E**). In the control group, daytime DBP was increased by 0.01 mmHg (95% CI: –2.41, 2.42) at the end of the study period, and there was no heterogeneity between the studies ($I^{2}$ = 0.0%, *p* = 0.940) (**Supplementary Fig. 4F**).

Nighttime SBP in the experimental groups was decreased by 0.29 mmHg compared with that in the control group (95% CI: –3.49, 2.90), but this was not significant. There was no heterogeneity between the studies ($I^{2}$ = 4.6%, *p* = 0.387) (**Supplementary Fig. 4G**). Nighttime SBP was decreased by 2.26 mmHg (95% CI: –5.02, 0.50) after treadmill exercise, and there was no heterogeneity between the studies ($I^{2}$ = 0.0%, *p* = 0.488) (**Supplementary Fig. 4H**). In the control group, nighttime SBP was decreased by 2.32 mmHg (95% CI: –5.71, 1.08) at the end of the study period, and there was no heterogeneity between the studies ($I^{2}$ = 0.0%, *p* = 0.742) (**Supplementary Fig. 4I**). Nighttime DBP in the experimental groups was decreased by 0.42 mmHg compared with that in the control group (95% CI: –2.70, 1.87), but this was not significant. There was no heterogeneity between the studies ($I^{2}$ = 0.0%, *p* = 0.873) (**Supplementary Fig. 4J**). Nighttime DBP was decreased by 1.57 mmHg (95% CI: –3.42, 0.28) after treadmill exercise, and there was no heterogeneity between the studies ($I^{2}$ = 0.0%, *p* = 0.814) (**Supplementary Fig. 4K**). In the control group, nighttime DBP was decreased by 1.22 mmHg (95% CI: –3.75, 1.31) at the end of the study period, and there was no heterogeneity between the studies ($I^{2}$ = 0.0%, *p* = 0.979) (**Supplementary Fig. 4L**).

Twenty-four-hour SBP in the experimental groups was decreased by 4.34 mmHg compared with that in the control group (95% CI: –9.40, 0.73), but this was not significant. There was no heterogeneity between the studies ($I^{2}$ = 57.8%, *p* = 0.069) (**Supplementary Fig. 4M**). Twenty-four-hour SBP was decreased by 4.96 mmHg (95% CI: –7.11, –1.01) after treadmill exercise, and there was no heterogeneity between the studies ($I^{2}$ = 45.8%, *p* = 0.136) (**Supplementary Fig. 4N**). In the control group, 24-hour SBP was increased by 0.12 mmHg (95% CI: –3.81, 4.06) at the end of the 24 hours, and there was no heterogeneity between the studies ($I^{2}$ = 0.0%, *p* = 0.781) (**Supplementary Fig. 4O**). Twenty-four-hour DBP in the experimental groups was decreased by 2.91 mmHg compared with that in the control group (95% CI: –5.60, –0.23). There was no heterogeneity between the studies ($I^{2}$ = 0.0%, *p* = 0.503) (**Supplementary Fig. 4P**). Twenty-four-hour DBP was decreased by 3.04 mmHg (95% CI: –5.41, –0.67) after treadmill exercise, and there was no heterogeneity between the studies ($I^{2}$ = 0.0%, *p* = 0.525) (**Supplementary Fig. 4Q**). In the control group, 24-hour DBP was decreased by 0.47 mmHg (95% CI: –3.28, 2.33) at the end of the 24 hours, and there was no heterogeneity between the studies ($I^{2}$ = 0.0%, *p* = 0.977) (**Supplementary Fig. 4R**).

### 3.5 Treatment Regimen: Walking

**Fig. 3.**
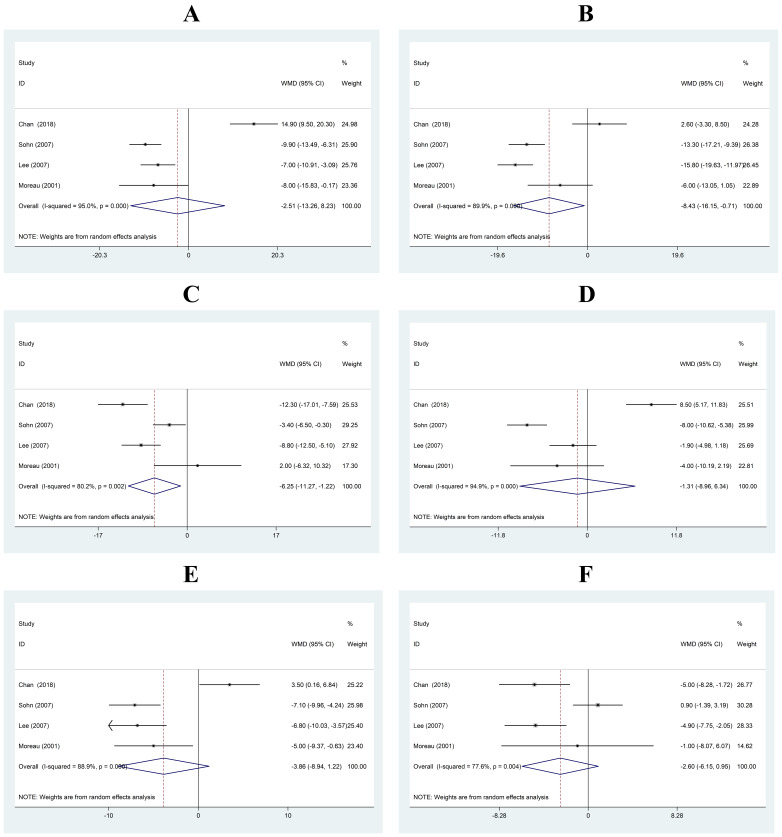
**Mean difference in SBP after walking**. (A) Changes in SBP (mmHg) between the experimental and control groups. (B) Changes in SBP (mmHg) in the experimental group. (C) Changes in SBP (mmHg) in the control group. (D) Changes in DBP (mmHg) between the experimental and control groups. (E) Changes in DBP (mmHg) in the experimental group. (F) Changes in DBP (mmHg) in the control group. Horizontal lines show 95% CIs with the point estimate at the center of the corresponding box. Within each subplot, boxes are proportional to the sample size from each study. Diamonds represent summary data centered on the pooled estimates, and their width spans the corresponding 95% CIs. CI, confidence interval; WMD, weighted mean difference; DBP, diastolic blood pressure; SBP, systolic blood pressure.

Five studies reported walking-related data [11, 13, 21, 22, 36] (Fig. 3). SBP in the experimental groups was decreased by 2.51 mmHg compared with that in the control group (95% CI: –13.26, 8.23), but this was not significant. There was heterogeneity between the studies ($I^{2}$ = 95.0%, *p* < 0.001) (Fig. 3A). SBP was decreased by 8.43 mmHg (95% CI: –16.15, –0.71) after the walking regimen, and there was heterogeneity between the studies ($I^{2}$ = 89.9%, *p* < 0.001) (Fig. 3B). In the control group, SBP was decreased by 6.25 mmHg (95% CI: –11.27, –1.22) at the end of the study period, and there was no heterogeneity between the studies ($I^{2}$ = 80.2%, *p* = 0.002) (Fig. 3C).

DBP in the experimental groups was decreased by 1.31 mmHg compared with that in the control group (95% CI: –8.960, –6.34), but this was not significant. There was heterogeneity between the studies ($I^{2}$ = 94.9%, *p* < 0.001) (Fig. 3D). DBP was decreased by 3.86 mmHg (95% CI: –8.94, 1.22) after the walking regimen, and there was heterogeneity between the studies ($I^{2}$ = 88.9%, *p* < 0.001) (Fig. 3E). In the control group, DBP was decreased by 2.60 mmHg (95% CI: –6.15, 0.95) at the end of the study period, and there was heterogeneity between the studies ($I^{2}$ = 77.6%, *p* = 0.004) (Fig. 3F).

### 3.6 Treatment Regimen: Resistance Training 

Two studies reported resistance training [38, 39] (**Supplementary Fig. 5**). SBP in the experimental groups was decreased by 5.08 mmHg compared with that in the control group (95% CI: –11.15, 1.00), but this was not significant. There was no heterogeneity between the studies ($I^{2}$ = 56.7%, *p* = 0.129) (**Supplementary Fig. 5A**). SBP was decreased by 3.33 mmHg (95% CI: –6.13, –0.53) after adopting resistance training, and there was no heterogeneity between the studies ($I^{2}$ = 0.0%, *p* = 0.415) (**Supplementary Fig. 5B**). Among the studies, the regimens of the control group were different and could not be combined.

DBP in the experimental groups was decreased by 1.88 mmHg compared with that in the control group (95% CI: –3.89, 0.13), but this was not significant. There was no heterogeneity between the studies ($I^{2}$ = 0.0%, *p* = 0.540) (**Supplementary Fig. 5C**). DBP was decreased by 1.65 mmHg (95% CI: –3.82, 0.53) after adopting resistance training, and there was no heterogeneity between studies ($I^{2}$ = 0.0%, *p* = 0.591) (**Supplementary Fig. 5D**). Among the studies, the regimens of the control group were different and could not be combined.

### 3.7 Treatment Regimen: Tai Chi 

Two studies reported Tai Chi [16, 36] (**Supplementary Fig. 6**). SBP in the experimental groups was decreased by 6.04 mmHg compared with that in the control group (95% CI: –37.40, 25.32), but this was not significant. There was heterogeneity between the studies ($I^{2}$ = 98.8%, *p* < 0.001) (**Supplementary Fig. 6A**). SBP was decreased by 9.14 mmHg (95% CI: –22.17, 3.89) after performing Tai Chi, and there was heterogeneity between the studies ($I^{2}$ = 93.2%, *p* < 0.001) (**Supplementary Fig. 6B**).

DBP in the experimental groups was decreased by 2.99 mmHg compared with that in the control group (95% CI: –21.02, 15.05), but this was not significant. There was heterogeneity between the studies ($I^{2}$ = 98.2%, *p* < 0.0001) (**Supplementary Fig. 6C**). DBP was decreased by 3.78 mmHg (95% CI: –13.58, 6.02) after performing Tai Chi, and there was heterogeneity between the studies ($I^{2}$ = 94.3%, *p* < 0.001) (**Supplementary Fig. 6D**). Among the studies, the regimens of the control group were different and could not be combined.

### 3.8 Treatment Regimen: Two to Three Types of Aerobic Exercise (Jogging + Walking + Biking/Calisthenics)

Two studies reported a variety of aerobic exercise regimens [12, 17] (**Supplementary Fig. 7**). SBP in the experimental groups was decreased by 2.49 mmHg compared with that in the control group (95% CI: –18.24, 13.26), but this was not significant. There was heterogeneity between the studies ($I^{2}$ = 91.0%, *p* = 0.001) (**Supplementary Fig. 7A**). SBP was decreased by 5.28 mmHg (95% CI: –11.98, 1.43) after adopting a variety of comprehensive aerobic exercise regimens, and there was no heterogeneity between the studies ($I^{2}$ = 54.0%, *p* = 0.141) (**Supplementary Fig. 7B**). In the control group, SBP was decreased by 3.95 mmHg (95% CI: –12.62, 4.71) after the regimen of the control group was adopted, and there was heterogeneity between the studies ($I^{2}$ = 78.2%, *p* = 0.032) (**Supplementary Fig. 7C**).

DBP in the experimental groups was decreased by 0.36 mmHg compared with that in the control group (95% CI: –2.57, 1.85), but this was not significant. There was no heterogeneity between the studies ($I^{2}$ = 0.0%, *p* = 0.330) (**Supplementary Fig. 7D**). DBP was decreased by 5.61 mmHg (95% CI: –7.71, –3.52) after adopting a variety of comprehensive aerobic exercise regimens, and there was no heterogeneity between the studies ($I^{2}$ = 0.0%, *p* = 0.845) (**Supplementary Fig. 7E**). DBP was decreased by 5.27 mmHg (95% CI: –7.40, –3.15) after the control program was adopted, and there was no heterogeneity between the studies ($I^{2}$ = 0.0%, *p* = 0.497) (**Supplementary Fig. 7F**).

## 4. Discussion

Exercise therapy has received increasing attention in recent years because of its advantage in effectively reducing BP, reducing the economic burden of using antihypertensive drugs and avoiding potential adverse side effects of drug treatment. The exercise attenuates both a rise in muscle sympathetic nerve activity (MSNA) and the blunting of neurovascular transduction (changes in blood pressure resulting from fluctuations in MSNA) [40], and this phenomenon may be explained by the increase in catecholamine clearance rate during moderate exercise, due to increased elimination from the tissues as a result of increased blood flow [41]. Meanwhile, regulating the imbalance of renin-angiotensin-aldosterone system homeostasis is one of the mechanisms by which exercise exerts cardiovascular protective effects. Eight weeks of swimming can upregulate the expression levels of angiotensin (1-7) and Mas receptor proteins in the left ventricle of spontaneously hypertensive rats [42]. Aerobic exercise can also regulate the production of reactive oxygen species (ROS) and nitric oxide (NO) in blood vessels by increasing the frequency and amplitude of hemodynamics and wall shear stress (WSS), improving endothelial dysfunction and NO bioavailability [43]. Furthermore, the exercise-induced reduction in muscle glycogen is a main contributor to this postexercise improvement in insulin sensitivity [44]. In brief, exercise therapy can lower BP by reducing sympathetic nerve excitability, adjusting the secretion level of human hormones, increasing insulin sensitivity, protecting and enhancing vascular function, inhibiting an overactivated renin–angiotensin aldosterone system, and reducing inflammatory factors [40, 41, 42, 43, 44, 45, 46, 47]. Previous studies have shown that a lack of physical activity leads to an increase in the prevalence of hypertension, while individuals who exercise or engage in physical activity reduce the risk of stroke, myocardial infarction, and cardiovascular mortality [48, 49].

At present, the normal recommendations to increase aerobics have been proposed in pertinent guidelines for preventing cardiovascular illnesses in China and internationally [6, 7]. The European Association of Preventive Cardiology and the Hypertension Council of the European Society of Cardiology have reached a consensus on a personalized exercise description for preventing and treating hypertension. They state that exercise lowers BP in hypertensive individuals, and recommend using a variety of exercise modes to achieve this goal [50]. The consensus points out that aerobics should be the primary physical activity for people with hypertension. In addition, different training methods, such as aerobics, isotonic training and isometric training, can lower SBP and DBP to different degrees. Therefore, the consensus emphasizes that a particular population should choose a certain exercise that maximizes the hypotensive effect in alignment with their own BP range, the BP level at baseline and prioritized selection. Personalized exercise prescriptions with the attributes of optimization of lifestyle interventions may be used to prevent and treat hypertension. However, the systematic review did not include the specific items of aerobics, including specific antihypertensive effects, such as fast walking, jogging, swimming, cycling, aerobics and rope skipping. Additionally, the daily use of neural exercise training forms, such as Tai Chi or yoga and heated pools, were excluded for some individuals with hypertension or prehypertension. However, in the clinical setting, the feasibility, accessibility and acceptability of different exercise modes in terms of different individuals are so different that, with a more personalized plan, BP can be lowered more effectively in the population.

Based on a systematic review and meta-analysis of RCTs on individuals with high-normal BP or hypertension who underwent different modes of exercise intervention, we found that varied exercise regimens had varying effects on lowering SBP, DBP and HR (**Supplementary Table 2**). We found the following: (1) Exercise in heated pools and moderate-intensity cycling were the most effective types of exercise in reducing SBP (45–60 minutes each time, three times a week) [20, 25, 33, 34]. (2) Treadmill exercise and IHG training were the most effective in reducing DBP (3–5 times a week, 30% of the maximum isometric voluntary contraction) [10, 14, 15, 18, 23, 24, 26, 27, 28, 30, 32, 35, 37, 38]. (3) The greatest reduction in HR was achieved by cycling [20, 25, 33] (45–60 minutes, three times a week).

This systematic review and meta-analysis showed that SBP was reduced after exercise in a heated pool and cycling [20, 25, 33, 34]. This study also showed that SBP was decreased after Tai Chi, but this was not significant [16, 36]. This result is in line with that of another study which showed that the Tai Chi group had lower BP than the Fast Walking group (SBP: –12.46 mmHg; DBP: –3.20 mmHg) [36]. This finding suggests that Tai Chi, which is a practical type of exercise, is beneficial for lowering BP and establishing a healthy lifestyle free from cardiovascular illness. Moreover, we found that DBP was reduced after treadmill exercise and IHG training [10, 14, 18, 23, 24, 26, 27, 28, 38]. To date, there have been few studies on how exercise affects HR. The current analysis suggested that cycling can decrease HR [20, 25, 33].

The effects of regular swimming and soccer on BP and vascular risk have received little attention. A study showed that SBP was decreased after 12 weeks of swimming training in sedentary older people [29]. Additionally, swimming significantly improved carotid artery compliance by 21% (*p* < 0.05). These findings suggest that swimming can help lower BP and improve blood vessel function. Another study showed that, in hypertensive middle-aged men, after 6 months of training, SBP significantly decreased from 151 ± 10 to 139 ± 10 mmHg and DBP from 92 ± 7 to 84 ± 6 mmHg (both *p* < 0.01) in the soccer training group (twice a week, 1 hour/time, n = 22, 68% received drug treatment) [31]. Additionally, the resting HR significantly declined by 8 ± 11 beats/minute (*p* < 0.05), and the arterial augmentation index dropped by 7.3 ± 14.0 (*p* < 0.05). These findings suggest that soccer training reduces BP and improves vascular function. Furthermore, another study analyzed the effect of 50%, 100% and 150% of the exercise amount recommended by the NIH Consensus Development Program on women’s health [19]. After 6 months, there was no apparent change in SBP or DBP in any groups. In summary, the effect of different forms of exercise on BP requires further investigation.

## 5. Conclusions

This systematic review and meta-analysis investigated the antihypertensive effects of various exercise regimens on HNBP and hypertensive populations. The included high-quality articles involving RCTs. This study shows that different exercise regimens have various effects on lowering SBP, DBP and HR as follows. (1) Swimming in heated pools and moderate-intensity cycling are the most effective types of exercise for reducing SBP. (2) Treadmill exercise and IHG training are the most effective exercises for reducing DBP. (3) Cycling is the most effective exercise for reducing HR. Our findings suggest that different types of exercise can effectively reduce the levels of SBP, DBP and HR in individuals with prehypertension or hypertension.

## Supplementary Material

Supplementary material associated with this article can be found, in the online version, at https://doi.org/10.31083/j.rcm2504117.
