# Supplementary material for: Exercise Prescription in Individuals with Prehypertension and Hypertension: Systematic Review and Meta-Analysis
Source: Rev Cardiovasc Med. 2024 Mar 27;25(4):117. doi: 10.31083/j.rcm2504117 (PMC11264040; doi:10.31083/j.rcm2504117)
Supplement: Supplementary file 1 [file 2153-8174-25-4-117-s1.zip › 2153-8174-25-4-117-s1/PRISMA_2020_checklist.pdf]

PRISMA 2020 checklist

Section and Topic	Item #	Checklist item	Page where item is reported (M = manuscript; S = supplements)
**TITLE**			
Title	1	Identify the report as a systematic review.	M1
**ABSTRACT**			
Abstract	2	See the PRISMA 2020 for Abstracts checklist.	M1
**INTRODUCTION**			
Rationale	3	Describe the rationale for the review in the context of existing knowledge.	M1
Objectives	4	Provide an explicit statement of the objective(s) or question(s) the review addresses.	M1
**METHODS**			
Eligibility criteria	5	Specify the inclusion and exclusion criteria for the review and how studies were grouped for the syntheses.	M2
Information sources	6	Specify all databases, registers, websites, organisations, reference lists and other sources searched or consulted to identify studies. Specify the date when each source was last searched or consulted.	M1, M2
Search strategy	7	Present the full search strategies for all databases, registers and websites, including any filters and limits used.	M1, M2
Selection process	8	Specify the methods used to decide whether a study met the inclusion criteria of the review, including how many reviewers screened each record and each report retrieved, whether they worked independently, and if applicable, details of automation tools used in the process.	M2<br>m
Data collection process	9	Specify the methods used to collect data from reports, including how many reviewers collected data from each report, whether they worked independently, any processes for obtaining or confirming data from study investigators, and if applicable, details of automation tools used in the process.	M1, M2
Data items	10	List and define all outcomes for which data were sought. Specify whether all results that were compatible with each outcome domain in each study were sought (e.g. for all measures, time points, analyses), and if not, the methods used to decide which results to collect.	M1, M2
Study risk of bias assessment	11	Specify the methods used to assess risk of bias in the included studies, including details of the tool(s) used, how many reviewers assessed each study and whether they worked independently, and if applicable, details of automation tools used in the process.	M2

Section and Topic	Item #	Checklist item	Page where item is reported (M = manuscript; S = supplements)
Effect measures	12	Specify for each outcome the effect measure(s) (e.g. risk ratio, mean difference) used in the synthesis or presentation of results.	M2
Synthesis methods	13a	Describe any methods used to explore possible causes of heterogeneity among study results (e.g. subgroup analysis, meta-regression).	M2
	13b	Describe any sensitivity analyses conducted to assess robustness of the synthesized results.	M2
Reporting bias assessment	14	Describe any methods used to assess risk of bias due to missing results in a synthesis (arising from reporting biases).	M2
Certainty assessment	15	Describe any methods used to assess certainty (or confidence) in the body of evidence for an outcome.	M2
**RESULTS**			
Study selection	16a	Describe the results of the search and selection process, from the number of records identified in the search to the number of studies included in the review, ideally using a flow diagram.	M2
	16b	Cite studies that might appear to meet the inclusion criteria, but which were excluded, and explain why they were excluded.	M2
Study characteristics	17	Cite each included study and present its characteristics.	M3
Risk of bias in studies	18	Present assessments of risk of bias for each included study.	M2
Results of individual studies	19	For all outcomes, present, for each study: (a) summary statistics for each group (where appropriate) and (b) an effect estimate and its precision (e.g. confidence/credible interval), ideally using structured tables or plots.	M2-M8 S2-S16
Results of syntheses	20a	Present results of all investigations of possible causes of heterogeneity among study results.	M2-M8 S2-S16
	20b	Present results of all sensitivity analyses conducted to assess the robustness of the synthesized results.	M2-M8 S2-S16
Reporting biases	21	Present assessments of risk of bias due to missing results (arising from reporting biases) for each synthesis assessed.	M2-8
**DISCUSSION**			
Discussion	23a	Provide a general interpretation of the results in the context of other evidence.	M8
	23b	Discuss any limitations of the evidence included in the review.	M9
	23c	Discuss any limitations of the review processes used.	M9

Section and Topic	Item #	Checklist item	Page where item is reported (M = manuscript; S = supplements)
	23d	Discuss implications of the results for practice, policy, and future research.	M9
**OTHER INFORMATION**			
Registration and protocol	24a	Provide registration information for the review, including register name and registration number, or state that the review was not registered.	M9
	24b	Indicate where the review protocol can be accessed, or state that a protocol was not prepared.	M9
Support	25	Describe sources of financial or non-financial support for the review, and the role of the funders or sponsors in the review.	M9
Competing interests	26	Declare any competing interests of review authors.	M9
