# Supplementary material for: Exercise Prescription in Individuals with Prehypertension and Hypertension: Systematic Review and Meta-Analysis
Source: Rev Cardiovasc Med. 2024 Mar 27;25(4):117. doi: 10.31083/j.rcm2504117 (PMC11264040; doi:10.31083/j.rcm2504117)
Supplement: Supplementary file 1 [file 2153-8174-25-4-117-s1.zip › 2153-8174-25-4-117-s1/Supplementary material.docx]

**Supplementary Fig. 1. The mean difference of SBP changes (mmHg) after treatment with heat pool.**

**Supplementary Fig. 2. The mean difference of BP and HR changes after treatment with cycling.**

**Supplementary Fig. 3. The mean difference of BP and HR changes after treatment with IHG.**

**Supplementary Fig. 4. The mean difference of 24 hours ambulatory blood pressure (ABPM) changes after treatment with treadmill.**

**Supplementary Fig. 5. The mean difference of SBP changes (mmHg) after treatment with resistance.**

**Supplementary Fig. 6. The mean difference of BP changes (mmHg) after treatment with Tai Chi.**

**Supplementary Fig. 7. The mean difference of SBP (mmHg) after doing 2-3 kinds of aerobic exercise in sync.**

**Supplementary Table 1. Basic characteristics of the included articles.**

**Supplementary Table 2. The changes of BP of RCT research in different exercise forms.**

**Supplementary methods: search terms**

**Supplementary Fig. 1. The mean difference of SBP changes (mmHg) after treatment with heat pool.** Horizontal lines show 95% CIs with the point estimate at the center of the corresponding box. Within each subplot, boxes are proportional to the sample size from each study. Diamonds represent summary data centered on the pooled estimates, and their width spans the corresponding 95% CIs. CI confidence interval, WMD weighted mean difference

A B

C D

E F

G H

I

**Supplementary Fig. 2. The mean difference of BP and HR changes after treatment with cycling.** (A) SBP Changes between experimental group and control group (mmHg). (B) SBP changes in experimental group (mmHg). (C) SBP changes in control group (mmHg). (D) DBP Changes between experimental group and control group (mmHg). (E) DBP changes in experimental group (mmHg). (F) DBP changes in control group (mmHg). (G) HR Changes between experimental group and control group (beats/min). (H) HR changes in experimental group (beats/min). (I) HR changes in control group (beats/min). Horizontal lines show 95% CIs with the point estimate at the center of the corresponding box. Within each subplot, boxes are proportional to the sample size from each study. Diamonds represent summary data centered on the pooled estimates, and their width spans the corresponding 95% CIs. CI confidence interval, WMD weighted mean difference

A B

C D

E F

G H

I

**Supplementary Fig. 3.** **The mean difference of BP and HR changes after treatment with IHG.** (A) SBP Changes between experimental group and control group (mmHg). (B) SBP changes in experimental group (mmHg). (C) SBP changes in control group (mmHg). (D) DBP Changes between experimental group and control group (mmHg). (E) DBP changes in experimental group (mmHg). (F) DBP changes in control group (mmHg). (G) HR Changes between experimental group and control group (mmHg). (H) HR changes in experimental group (beats/min). (I) HR changes in control group (beats/min). Horizontal lines show 95% CIs with the point estimate at the center of the corresponding box. Within each subplot, boxes are proportional to the sample size from each study. Diamonds represent summary data centered on the pooled estimates, and their width spans the corresponding 95% CIs. CI confidence interval, WMD weighted mean difference, IHG isometric handgrip training.

A B

**** ****

C D

E F

G H

**** ****

I J

K L

M N

O P

Q R

**Supplementary Fig. 4. The mean difference of 24 hours ambulatory blood pressure (ABPM) changes after treatment with treadmill.** (A) dSBP Changes between experimental group and control group (mmHg). (B) dSBP changes in experimental group (mmHg). (C) dSBP changes in control group (mmHg). (D) dDBP Changes between experimental group and control group (mmHg). (E) dDBP changes in experimental group (mmHg). (F) dDBP changes in control group (mmHg). (G) nSBP Changes between experimental group and control group (mmHg). (H) nSBP changes in experimental group (mmHg). (I) nSBP changes in control group (mmHg). (J) nDBP Changes between experimental group and control group (mmHg). (K) nDBP changes in experimental group (mmHg). (L) nDBP changes in control group (mmHg). (M) 24hSBP Changes between experimental group and control group (mmHg). (N) 24h SBP changes in experimental group (mmHg). (O) 24h SBP changes in control group (mmHg). (P) 24hDBP Changes between experimental group and control group (mmHg). (Q) 24h DBP changes in experimental group (mmHg). (R) 24h DBP changes in control group (mmHg). Horizontal lines show 95% CIs with the point estimate at the center of the corresponding box. Within each subplot, boxes are proportional to the sample size from each study. Diamonds represent summary data centered on the pooled estimates, and their width spans the corresponding 95% CIs. CI confidence interval, WMD weighted mean difference, dSBP daytime SBP, dDBP daytime DBP, nSBP nighttime SBP, nDBP nighttime DBP, 24h SBP 24-h ambulatory systolic blood pressure, 24h DBP 24-h ambulatory diastolic blood pressure.

A B

**** ****

C D

**** ****

**Supplementary Fig. 5. The mean difference of SBP changes (mmHg) after treatment with resistance.** (A) SBP Changes between experimental group and control group (mmHg). (B) SBP changes after resistance (mmHg). (C) DBP changes between experimental group and control group (mmHg). (D) DBP changes after resistance (mmHg). Horizontal lines show 95% CIs with the point estimate at the center of the corresponding box. Within each subplot, boxes are proportional to the sample size from each study. Diamonds represent summary data centered on the pooled estimates, and their width spans the corresponding 95% CIs. CI confidence interval, WMD weighted mean difference.

A B

**** ****

C D

**Supplementary Fig. 6. The mean difference of BP changes (mmHg) after treatment with Tai Chi.** (A) SBP Changes between experimental group and control group (mmHg). (B) SBP changes after Tai Chi (mmHg). (C) DBP Changes between experimental group and control group (mmHg). (D) DBP changes (mmHg) after Tai Chi. Horizontal lines show 95% CIs with the point estimate at the center of the corresponding box. Within each subplot, boxes are proportional to the sample size from each study. Diamonds represent summary data centered on the pooled estimates, and their width spans the corresponding 95% CIs. CI confidence interval, WMD , weighted mean difference.

A B

C D

E F

**Supplementary Fig. 7. The mean difference of SBP (mmHg) after doing 2-3 kinds of aerobic exercise in sync.** (A) SBP Changes between experimental group and control group (mmHg). (B) SBP changes in experimental group (mmHg). (C) SBP changes in control group (mmHg). (D) DBP Changes between experimental group and control group (mmHg). (E) DBP changes in experimental group (mmHg). (F) DBP changes in control group (mmHg). Horizontal lines show 95% CIs with the point estimate at the center of the corresponding box. Within each subplot, boxes are proportional to the sample size from each study. Diamonds represent summary data centered on the pooled estimates, and their width spans the corresponding 95% CIs. CI confidence interval, WMD weighted mean difference.

**Supplementary Table 1. Basic characteristics of the included articles.**

|  | Randomization^a^ | Concealment of allocation^b^ | Double blinding^c^ | Withdrawals and dropouts^d^ | Jadad score^e^ |
| --- | --- | --- | --- | --- | --- |
| Blumenthal, et al. 2000^10^ | 1 | 0 | 0 | 1 | 2 |
| Cooper, et al. 2000^11^ | 2 | 2 | 0 | 1 | 5 |
| Georgiades, et al. 2000^12^ | 1 | 0 | 0 | 1 | 2 |
| Moreau, et al. 2001^13^ | 1 | 0 | 2 | 0 | 3 |
| Tsai, et al. 2002^14^ | 1 | 0 | 0 | 1 | 2 |
| Taylor, et al. 2003^15^ | 1 | 0 | 0 | 0 | 1 |
| Tsai, et al. 2003^16^ | 2 | 2 | 2 | 1 | 7 |
| Tsuda, et al. 2003^17^ | 1 | 0 | 0 | 0 | 1 |
| Tsai, et al. 2004^18^ | 1 | 0 | 2 | 1 | 4 |
| Church, et al. 2007^19^ | 2 | 2 | 2 | 1 | 7 |
| Laterza, et al. 2007^20^ | 1 | 0 | 0 | 0 | 1 |
| Lee, et al. 2007^21^ | 2 | 2 | 2 | 1 | 7 |
| Sohn, et al. 2007^22^ | 2 | 1 | 2 | 0 | 5 |
| Westhoff, et al. 2007^23^ | 1 | 0 | 2 | 1 | 4 |
| Guimaraes, et al. 2010^24^ | 2 | 2 | 2 | 1 | 7 |
| Lamina, et al. 2010^25^ | 1 | 0 | 2 | 0 | 3 |
| Waib, et al. 2011^26^ | 1 | 0 | 0 | 1 | 2 |
| Dimeo, et al. 2012^27^ | 1 | 0 | 0 | 1 | 2 |
| Molmen, et al. 2012^28^ | 2 | 1 | 0 | 1 | 4 |
| Nualnim, et al. 2012^29^ | 0 | 0 | 0 | 1 | 1 |
| Badrov, et al. 2013^30^ | 1 | 0 | 0 | 1 | 2 |
| Krustrup, et al. 2013^31^ | 1 | 0 | 0 | 0 | 1 |
| Millar, et al. 2013^32^ | 1 | 0 | 0 | 1 | 2 |
| Arca, et al. 2014^33^ | 1 | 0 | 0 | 0 | 1 |
| Guimaraes, et al. 2014^34^ | 1 | 2 | 2 | 1 | 6 |
| Pagonas, et al. 2017^35^ | 2 | 1 | 2 | 1 | 6 |
| Chan, et al. 2018^36^ | 2 | 2 | 2 | 1 | 7 |
| Ogbutor, et al. 2019^37^ | 2 | 2 | 0 | 0 | 4 |
| Boeno, et al. 2020^38^ | 2 | 2 | 2 | 1 | 7 |
| Son, et al. 2020^39^ | 2 | 2 | 2 | 1 | 7 |

1. Randomization: the method of randomization is described appropriately (2 points); the randomized trials did not describe the methods of random assignment (1 point); not randomized or inappropriate method of randomization (0 points).
2. Conealment of allocation: the method of concealment of allocation is described appropriately (2 points); the study only indicated the use of a random number table or other random assignment methods (1 point), not preventing the predictability of grouping (0 points).
3. Double-blinding: the randomization method is described appropriately (2 points); the study only stated double-blinding, but the methods were not described (1 point); not double-blind or inappropriate blinding (0 points).
4. Withdrawals and dropouts: a description of the numbers and reasons for withdrawals and dropouts (1 point); not follow-up (0 points).
5. The total Jadad score of 1–3 is considered low-quality studies, and the score of 4–7 is considered high-quality studies.

**Supplementary Table 2. The changes of BP of RCT research in different exercise forms.**

| **Exercise Type** | **SBP changes (95% CI) (mmHg)** | | **DBP changes (95% CI) (mmHg)** | | **HR changes (95% CI) (beats/min)** | |
| --- | --- | --- | --- | --- | --- | --- |
|  | Experimental Group | Control Group | Experimental Group | Control Group | Experimental Group | Control Group |
| Heat pool[33,34] | -15.62(-23.83,-7.41) | - | - | - | - | - |
| Cycling [20,25,33] | -14.76(-17.04,-12.48) | 2.55(-0.01,5.11) | -5.07(-9.17,-0.98) | -1.07(-1.48,-0.66) | -9.57(-11.25,-7.90) | -1.61(-5.52,2.31) |
| IHG(isometric handgrip training) [15,30,32,35,37] | -8.08(-12.97,-3.20) | -3.27(-10.22,3.68) | -5.57(-7.48,-3.66) | -0.04(-5.09,5.01) | -1.37(-6.32,3.58) | 1.62(-2.64,5.87) |
| Treadmill[10,14,18,23,24,26,27,28,38] | - -9.43(-11.78,-7.08) - dSBP:-5.58(-8.33,-2.82) - nSBP:-2.26(-5.02，0.50) - 24h SBP:-4.06(-7.11,-1.01) | - -2.56(-5.34,0.23) - dSBP:0.80(-2.22,3.82) - nSBP:-2.32(-5.71,1.08) - 24h SBP:0.12(-3.81,4.06) | - -5.16(-6.78,-3.55) - dDBP:-4.26(-6.18,-2.34) - nDBP:-1.57(-3.42,0.28) - 24h DBP:-3.04(-5.41,-0.67) | - 1.50(-0.14,3.15) - dDBP:0.01(-2.41,2.42) - nDBP:-1.22(-3.75,1.31) - 24h DBP:-0.47(-3.28,2.33) | -2.18(-4.20,-0.16) | 0.25(-1.75,2.25) |
| Walking[11,13,21,22,36] | -8.43(-16.15,-0.71) | -6.25(-11.27,-1.22) | -3.86(-8.94,1.22) | -2.60(-6.15,0.95) | - | - |
| Resistance[38,39] | -3.33(-6.13,-0.53) | - | -1.65(-3.82,0.53) | - | - | - |
| Tai Chi [16,36] | -9.14(-22.17,3.89) | - | -3.78(-13.58,6.02) | - | - | - |
| 2-3 kinds of aerobic exercise in sync [12，17] | -5.28(-11.98,1.43) | -3.95(-12.62,4.71) | -5.61(-7.71,-3.52) | -5.27(-7.40,-3.15) |  |  |

**Supplementary methods: search terms**

**Embase.com**

**#1. Exercise*:ti**

**#2. ‘Physical activity’:ti OR ‘physically active’:ti OR ‘physical activities’:ti**

**#3. Training*:ti AND (aerobic*:ti,ab OR strength*:ti,ab OR physical:ti,ab OR endurance:ti,ab OR resistance:ti,ab)**

**#4. Sports:ti**

**#5. #1 OR #2 OR #3 OR #4**

**#6. Hypertension:ti OR hypertensive:ti OR hypertens*:ti OR ‘blood pressure*’:ti OR ‘prehypertension’:ti**

**#7. ‘Systematic review’/exp**

**#8. ‘Meta analysis’/exp**

**#9. ‘Systematic review’:ti OR ‘meta-analysis’:ti OR metaanalysis:ti OR systematic:ti**

**#10. #7 OR #8 OR #9**

**#11. #5 AND #6 AND #10**

**#12. ‘Diet therapy’/exp**

**#13. #11 NOT #12**
